# Institutional Notes and News

**Published:** 1910-06-25

**Authors:** 


### Institutional Notes and News.

**Crewkerne Hospital, Somerset.**

A piece of good fortune, or rather the inevitable result of energy and public spirit, has attended the finances of the Crewkerne Hospital in the last twelve months, by which a debt of over £48 has been reduced by £29 to the much more manageable sum of £19. The increase of income which accomplished this was due to £50 which came from football, a hospital football cup having been given by Mr. Dyson (who presided at the annual meeting), and to £14 which was the proceeds of the local Marathon race. The committee thanked all those by whose personal energies the arrangements produced this satisfactory result, and special mention was made of Mr. G. F. Gosney, the secretary, who was complimented by Mr. Dyson for his enterprise and hard work. The ward cases for the year numbered 121, and in the out-patient department attached to the hospital 57 persons and casualty cases had been attended. Thus a year of progress has been completed which should encourage all supporters of the institution to make the fear of getting into debt a thing of the past. Matters other than financial are noted in our Report (p. 388).

**Addenbrooke's Hospital, Cambridge.**

The new electrical department at Addenbrooke's Hospital, Cambridge, has been opened, chiefly owing to the generosity of Mrs. Lilley Smith, who started the fund with £50, or approximately one-fifth of the sum needed. Another £50 was given by the Borough Education Committee. Apart from the search table and the motor generator the room is fitted with electrical apparatus for galvanisation and for taking stereoscopic photographs. The department will be under the control of Dr. Shillington Scales and Mr. Field. Colonel T. W. Harding, who presided at the opening, said that though the electrical department was expensive no modern hospital could do without it. He believed that the County Council would follow the example of the borough on behalf of the school children, whose medical inspection added to the hospital work, and consent to make an annual grant for the upkeep of the department. It is hoped that the initial expenditure of installing the *x*-ray apparatus will be paid off without any raid on the general funds.

**East Suffolk and Ipswich Hospital.**

The supporters of the East Suffolk and Ipswich Hospital have just had the opportunity of inspecting the improvements which their help has made it possible to carry out. These consist of, roughly, three blocks of buildings; one on the north of the central group, which is the administration block; another, on the north-west, which is the isolation block; and a third which contains the mortuary and laundry and runs along the north-east side of the property. Besides this, however, the old buildings have been renovated internally—for instance, an electric lift has been installed and a new operation unit has been added. Dr. J. H. Bartlett, the President, received the large crowd of subscribers who attended to inspect these improvements, and Mr. Herbert Mason, who presided, gave an outline of the gradual modernisation of the hospital from the date of Queen Victoria's Jubilee, when the first new wing was built. The present alterations have owed much to the attention, care, and time which Mr. E. P. Ripley has bestowed upon them. The original plan was to spend £12,000, for which the richer classes were appealed to, but ultimately the scheme grew, the committee feeling that the present opportunity should be used to complete the improvements needed, and this has meant the spending of some £20,000, to which the working classes have contributed most generously and loyally their share. Mr. E. A. Oliver, their representative at the opening, said that the working-men had contributed £900 in 1908, but that he hoped the present year would see this raised to £1,500.

**Philipson Children's Sanatorium.**

Two years ago the Philipson Children's Santorium, at Stannington, near Newcastle, was opened for young tuberculous patients of the poor. The original scheme provided for the building of two wings which would accommodate ninety patients, but want of money limited the sanatorium to one wing only, which, since the opening, has received 200 patients. No immediate prospect of completing the original design was anticipated at the recent annual meeting, but since then Sir W. H. Stephenson, Lord Mayor of Newcastle, who took the chair on that occasion, has come forward and placed £4,000 at the Committee's disposal to build and equip the second wing. This wing will be christened "Lady Stephenson" as a mark of gratitude to the donor. It is proposed to place the new building at the disposal of those children who suffer from local tuberculosis, and whose cases are hardly eligible for sanatorium treatment in most institutions, and who therefore have been deprived of the advantages open to patients tuberculous in the more popular sense of the word. It is obvious at once that this new wing will increase, if not double, the expenses of maintenance, and the Hon. Secretary, Mr. J. H. Watson, hopes that the public will realise this; for if fresh annual subscriptions are not forthcoming, Sir W. H. Stephenson's generous gift may be deprived of its usefulness, and, unless the public intelligence be awakened, almost prove an incubus to the sanatorium. No Newcastle citizen can consent to allow his Lord Mayor's public spirit to be robbed of its fruitfulness by want of annual support.

**Morningfield Hospital, Aberdeen.**

Mr. George M. Cook presided over the recent annual meeting of the Morningfield Hospital, Aberdeen. The report, which was read by Mr. Patrick Cooper, the secretary, stated that the average number of patients in the hospital throughout the year was 78, and the average cost per patient was £25, or £4 less than it was six years ago. Twelve applications for admission were found eligible, but were refused through want of room. The ordinary income, including the patients' contributions, was £1,462, and the ordinary expenditure £2,082, leaving the hospital with a debt of £620 on the current year's accounts. This, however, was met by the diversion of legacies from their true goal, the capital account, with the exception of £34, which still remained unpaid for. The chairman, who has been in office since 1904, and therefore retires by rotation, drew attention to the point where economy meant privation to the patients, and said how necessary it was that their comfort should be safeguarded, which the present cost per patient he thought provided, without admitting extravagance in any way. He pointed out that the relative expense of the garden was compensated for by the vegetables and fruit, which supplied from it all the hospital needs. Professor Davidson, in seconding the adoption of the report, which was carried, emphasised the advantage to an institution of a host of small subscribers over the few large givers whose support might be withdrawn at any time.

